# Exosome‐transferred long non‐coding RNA ASMTL‐AS1 contributes to malignant phenotypes in residual hepatocellular carcinoma after insufficient radiofrequency ablation

**DOI:** 10.1111/cpr.12795

**Published:** 2020-07-28

**Authors:** Dening Ma, Xinyi Gao, Zhuo Liu, Xingang Lu, Haixing Ju, Ning Zhang

**Affiliations:** ^1^ Institute of Cancer and Basic Medicine (ICBM) Chinese Academy of Sciences 1 Banshan East Road Hangzhou 310022 China; ^2^ Department of Colorectal Surgery Cancer Hospital of the University of Chinese Academy of Science 1 Banshan East Road Hangzhou 310022 China; ^3^ Department of Colorectal Surgery Zhejiang Cancer Hospital 1 Banshan East Road Hangzhou 310022 China; ^4^ Department of Radiology Cancer Hospital of the University of Chinese Academy of Sciences 1 Banshan East Road Hangzhou 310022 China; ^5^ Department of Radiology Zhejiang Cancer Hospital 1 Banshan East Road Hangzhou 310022 China; ^6^ Department of Hepatic Surgery Fudan University Shanghai Cancer Center Cancer Hospital 270 Dong-An Road Shanghai 200032 China

**Keywords:** ASMTL‐AS1, exosomes, HCC, NLK, YAP nuclear translocation

## Abstract

**Objectives:**

Long non‐coding RNAs (lncRNAs) are emerging RNA regulators in cancer progression, including in hepatocellular carcinoma (HCC). Recently, insufficient radiofrequency ablation (RFA) has been reported to lead to recurrence and metastasis of residual HCC tumours. Herein, we aimed to the role of ASMTL‐AS1 in residual HCC after insufficient RFA.

**Materials and methods:**

In vitro insufficient RFA model was simulated in Huh7 cells and subsequently named Huh7‐H cells. In vitro and in vivo assays were conducted to investigate ASMTL‐AS1 function in HCC.

**Results:**

LncRNA ASMTL‐AS1 low expressed in normal human liver was found to be highly expressed in HCC tissues and further increased in tumours after insufficient RFA. ASMTL‐AS1 expression was related to stage, metastasis and prognosis in HCC. Huh7‐H possessed higher ASMTL‐AS1 level and more aggressive than Huh7 cells. ASMTL‐AS1 contributed to the malignancy of HCC cells both in vitro and in vivo. Mechanistically, ASMTL‐AS1 was trans‐activated by MYC and promoted NLK expression to activate YAP signalling via sequestering miR‐342‐3p in HCC. Interestingly, ASMTL‐AS1 could be wrapped by exosomes and then convey malignancy through NLK/YAP axis between cells even in residual HCC after insufficient RFA.

**Conclusions:**

Exosomal ASMTL‐AS1 aggravates the malignancy in residual HCC after insufficient RFA via miR‐342‐3p/NLK/YAP signalling, opening a new road for the treatment of HCC and the prevention of recurrence or metastasis of residual HCC after insufficient RFA.

## 1 INTRODUCTION

Hepatocellular carcinoma (HCC), which always results in high morbidity and mortality, is one of the most aggressive cancers all over the world.^1^ In China, HCC ranks third in terms of the leading causes of cancer‐related death.^2^ Although liver transplantation is the preferred treatment for HCC patients, its application usually be limited due to deficiency of good donor organs.^3^ In this case, surgical resection and radiofrequency ablation (RFA) are extensively adopted to cure this disease.^4^ However, the option of RFA or surgery depends on the specific conditions of patients.^5^ Disappointingly, clinical studies indicated that sufficient ablation is hardly achieved in HCC patients,^6^ which might be attributed to insufficient ablative margin,^7^ heat loss in tumours via vessels^8^ or a low‐temperature area in a large tumour when ablation.^9^ More seriously, residual tumours after insufficient RFA always lead to recurrence and metastasis.^10^ Hence, understanding the molecular mechanism through which residual tumours after insufficient RFA contributed to tumorigenesis and metastasis is the urgent work for researchers.

In past few decades, long non‐coding RNAs (lncRNAs), defined as a subclass of non‐coding RNAs (without protein‐coding ability) with more than 200 nts in length,^11^ have been increasingly recognized to be involved in cancer development^12^ including HCC.^13^ As an example, lncRNA CASC9 facilitates HCC proliferation by interacting with HNRNPL to regulate AKT signalling.^14^ However, the significance of lncRNAs in the development of residual HCC after insufficient RFA has hardly been discussed yet, except a previous report which illustrated that lncRNA FUNDC2P4 suppresses epithelial‐mesenchymal transition (EMT) by abrogating E‐cadherin expression in residual HCC after insufficient RFA.^15^ Acetylserotonin O‐methyltransferase like (ASMTL) antisense RNA 1 (ASMTL‐AS1) is a newly found lncRNA that locates at Xp22.33 and Yp11.2. Nevertheless, the function of ASMTL‐AS1 has nearly unexplored in any disease before, let alone cancers like HCC.

Exosomes originated from endosomes are nanoscale membrane vesicles (with a size of 30 ~ 100 nm) co‐existing with microvesicles and apoptotic bodies in the extracellular microenvironment.^16^ Exosomes can mediate the communication among cancer cells or between tumours and non‐tumours by transferring a variety of proteins, DNAs and RNAs (including lncRNAs).^17^ In HCC, exosomes have also been revealed to serve important roles during tumour development and progression, especially drug resistance and metastasis. For example, lncRNA FAL1 transferred by exosomes can increase HCC cell proliferation and migration.^18^ Linc‐VLDLR within exosomes regulates the response of HCC cells to chemotherapy.^19^ Exosomal microRNA‐103 derived from HCC cells promotes metastasis through modulating targeting junction proteins.^20^ However, whether exosomes also participate in the development and metastasis of residual HCC after insufficient RFA remains largely unknown.

Interestingly, LncBook (https://bigd.big.ac.cn/lncbook/index) and NONCODE (http://www.noncode.org/) indicated ASMTL‐AS1 is low or even un‐expressed in normal human liver tissues. On the contrary, starBase suggested a potential up‐regulation tendency (although not significant, which may due to limited samples and individual differences) in liver hepatocellular carcinoma (LIHC) tissues compared with normal tissues. On this basis, we suspected that ASMTL‐AS1 might be involved in HCC development. More intriguingly, lncLocator (http://www.csbio.sjtu.edu.cn/bioinf/lncLocator/) predicted ASMTL‐AS1 might mainly exist in exosomes of cells, which implied an involvement of exosomes during cancer progression. In this study, according to above data, we probed into the role of ASMTL‐AS1 in residual HCC after insufficient RFA and whether exosomes were also implicated in to convey ASMTL‐AS1 between HCC cells.

## 2 MATERIALS AND METHODS

### 2.1 Tissue samples

Human HCC tissues and matched para‐tumour samples were collected from 70 HCC patients undergoing the surgical resection, while 28 of 70 were residual tumours obtained from HCC patients with insufficient RFA treatment. All tissue samples were maintained in liquid nitrogen at −80°C and used with the informed consents from all subjects and the approval from the Ethics Committee of Institute of Cancer Research and Basic Medical Sciences of Chinese Academy of Sciences, Cancer Hospital of University of Chinese Academy of Sciences.

### 2.2 Cell lines

Human HCC cell lines (Hep2G, Huh7, HCCLM3 and SMMC7721) and normal human hepatic cell line (THLE‐2) were available from the Chinese Academy of Sciences (Shanghai, China), maintained in 5% CO_2_ at 37°C. RPMI 1640 medium (Gibco) was applied for culturing cells with 10% foetal bovine serum (FBS; Gibco) and 1% pen/strep. To mimic in vitro cancerous cells after insufficient RFA, Huh7 cells in 7 cm diameter petri dishes were placed at 1 × 10^5^ cells per well for 24 hours, blocked with parafilm and submerged into 47°C water bath. Following incubation under normal condition, confluent cells were heated in 47°C water bath, and surviving cells were named as Huh7‐H.

### 2.3 RNA extraction and QRT‐PCR

Total RNA was extracted using TRIzol reagent (Thermo Fisher Scientific) as per user guidebook, denatured and reversely transcribed into cDNA using Reverse Transcription Kit (TaKaRa). qRT‐PCR was carried out by BioRad SYBR Green Super Mix (Bio‐Rad), with GAPDH or U6 as internal control. Relative expression levels of genes were calculated by the 2^−∆∆Ct^ method.

### 2.4 EdU incorporation assay

Cells in each group were planted in 96‐well plates for incubating with EdU incorporation assay kit (Ribobio) as instructed by manufacturer. Cells were observed after adding DAPI solution under fluorescent microscope (Leica).

### 2.5 Colony formation assay

Clonogenic cells at logarithmic growth phase were planted in 6‐well plates for 14‐day culture process. After fixation in 4% paraformaldehyde, cells were processed with 0.1% crystal violet. Colonies were counted.

### 2.6 Transwell assay

Transwell assays were conducted using 8 μm pore transwell insert (Millipore) with or without Matrigel for invasion or migration. 2 × 10^4^ cells in serum‐free medium were seeded into upper chamber. Lower chamber was filled with 700 μL of complete medium. Forty‐eight hours later, the migrated or invaded cells were fixed for 20 minutes and stained with crystal violet for 10 minutes. Number of cells was determined as the mean of cell number in 5 random filed using microscopy.

### 2.7 Western blot

Proteins lysates obtained in RIPA lysis buffer were subjected to 10% SDS‐PAGE and transferred onto the PVDF membrane. Membranes were sealed with 5% skimmed milk and incubated with specific primary antibodies (Abcam) overnight at 4°C. Followed by washing in TBST, secondary antibodies conjugated with HRP were used. Proteins were visualized using a detection system of enhanced chemiluminescence (ECL). GAPDH served as the internal reference.

### 2.8 Plasmid transfection

The pcDNA3.1 vectors and NC vectors were available from Genepharma Company to overexpress ASMTL‐AS1, MYC and NLK in cell samples using transfection kit Lipofectamine 2000 (Invitrogen). The miR‐342‐3p mimics and miR‐NC, as well as the shRNAs specific to ASMTL‐AS1, MYC and NC shRNAs, were also acquired from Genepharma Company. Forty‐eight hours later, cells were reaped from each group for analysis.

### 2.9 Animal study

Animal study was approved by the Institutional Animal Care and Use Committee of Institute of Cancer Research and Basic Medical Sciences of Chinese Academy of Sciences, Cancer Hospital of University of Chinese Academy of Sciences. Male BALB/c‐nu mice (6‐8 weeks of age), available from Vital River, were injected subcutaneously with 1 × 10^7^ transfected cells, with tumour volume monitored every 4 days. Twenty‐eight days later, mice were sacrificed and weighed. For in vivo liver metastasis, transfected cells were injected into mice through tail veins. The metastatic nodules were analysed by H&E staining.

### 2.10 In situ hybridization

The tissue sections were dehydrated in ethanol to hybridize with 40 nmol/L of specific RNA in situ hybridization (ISH) probes for 1 hour and then rinsed in PBS. After nuclear counterstaining with DAPI, RNA level was assayed by microscope.

### 2.11 Immunohistochemistry

Tissue samples from animal study were fixed by 4% paraformaldehyde and embedded in paraffin for cutting. The successive sections (4 μm) were acquired and cultured with specific antibodies (Abcam) for immunohistochemistry (IHC) analysis.

### 2.12 Chromatin immunoprecipitation assay

Cells were fixed for 20 minutes for forming the DNA and protein cross‐linking, then fragmented by ultrasonic and immunoprecipitated with anti‐MYC antibody or control IgG antibody (Abcam). 30 μL magnetic beads were then added for 2 hours, and precipitated DNA was purified for qRT‐PCR.

### 2.13 Luciferase reporter assay

For ASMTL‐AS1 promoter luciferase assay, HEK‐293T cells were co‐transfected with the pGL3‐vector containing ASMTL‐AS1 promoter, pRL‐TK‐Renilla (Promega) and the MYC overexpression or silencing plasmids. Forty‐eight hours later, luciferase activity was measured using the Dual‐Luciferase Reporter Assay System (Promega). Besides, the ASMTL‐AS1 or NLK fragment covering the wild‐type or mutated miR‐342‐3p binding sites was inserted to pmirGLO vector (Promega), termed ASMTL‐AS1‐WT/MUT and NLK‐WT/MUT for luciferase analysis.

### 2.14 Fluorescence in situ hybridization analysis

The ASMTL‐AS1‐specific fluorescence in situ hybridization analysis (FISH) probe was available from Ribobio and employed as per the user guide. Cell nucleus was analysed by DAPI staining using microscope.

### 2.15 RNA immunoprecipitation assay

RNA immunoprecipitation (RIP) assay was implemented as guided by the protocol of EZ‐Magna RIP RNA Binding Protein Immunoprecipitation Kit (Millipore). Antibodies against Ago2 and control IgG were acquired from Abcam. Precipitated RNAs were collected by adding beads and measured via qRT‐PCR.

### 2.16 Co‐immunoprecipitation assay

Cells were processed with IP lysis buffer to acquire cell lysates and then mixed with specific antibodies in constant speed at 4°C. Normal IgG served as control. Then, the mixture was cultured with beads for 2 hours, rinsed in IP lysis buffer and then eluted for Western blot analysis.

### 2.17 Subcellular fractionation

Subcellular fractionation assay was conducted to acquire the cell nuclei and cell cytoplasm fractions using PARIS™ Kit (Ambion) in line with the standard method. The isolated RNAs were monitored by Western blot method.

### 2.18 Immunofluorescence

After cells were adhered to the culture slides, cells in PBS were blocked in 5% BSA for probing with primary antibodies for 1.5 hours at room temperature. After washing, the secondary antibodies were added for 40 minutes. Cells were finally treated with DAPI and visualized by fluorescent microscope.

### 2.19 Isolation and identification of exosomes

Exosomes in Huh7 and Huh7‐H cell lines were isolated in line with the instructions of the Exosomes Isolation Kit (Thermo Fisher Scientific). Cells were cultured with 10% depleted FBS that was pre‐depleted of bovine exosomes via ultracentrifugation at 4°C for 16 hours. Confluent cells were rinsed three times in PBS with 10% depleted FBS for 48 hours. Exosomes were isolated and purified from Huh7 or Huh7‐H cell supernatant, named as Huh7‐Exos and Huh7‐H‐Exos.

### 2.20 Exosome morphology and size analysis

The morphology of exosomes was observed under transmission electron microscopy (TEM). Exosomes derived from each group were visualized and photographed with digital camera (Olympus). The size and number of exosomes were measured and determined using nanoparticle tracking analysis (NTA) measurements and tunable resistive pulse sensing (TRPS, IZON qNano).

### 2.21 Statistical analyses

Data were expressed as means ± SD (standard deviation) from three independent bio‐repeats. Overall survival was estimated by Kaplan‐Meier analysis. Correlation analysis was examined by Pearson's chi‐squared analysis. Statistical difference was calculated with Student's *t* test or one‐way analysis of variance (ANOVA) using SPSS 19.0 (SPSS), with *P* < .05 as threshold.

## 3 RESULTS

### 3.1 Up‐regulation of ASMTL‐AS1 enhances the malignancy of residual HCC cells after insufficient RFA

In this research, we studied a novel lncRNA ASMTL‐AS1 which had never been explored yet. First of all, we were curious about the characteristics of ASMTL‐AS1. Normally, ASMTL‐AS1 was suggested by LncBook and NONCODE to be low expressed and even nearly deficient in human liver tissues (Figure S1A,B). By contrast, ASMTL‐AS1 expression tended to be up‐regulated in liver hepatocellular carcinoma (LIHC) tissues relative to the normal ones (Figure S1C). Also, the results from LncBook showed that ASMTL‐AS1 had no protein‐coding ability by three prediction tools (Figure S1D). In this case, we were strongly interested in the potential role of ASMTL‐AS1 in HCC.

**Figure 1:**
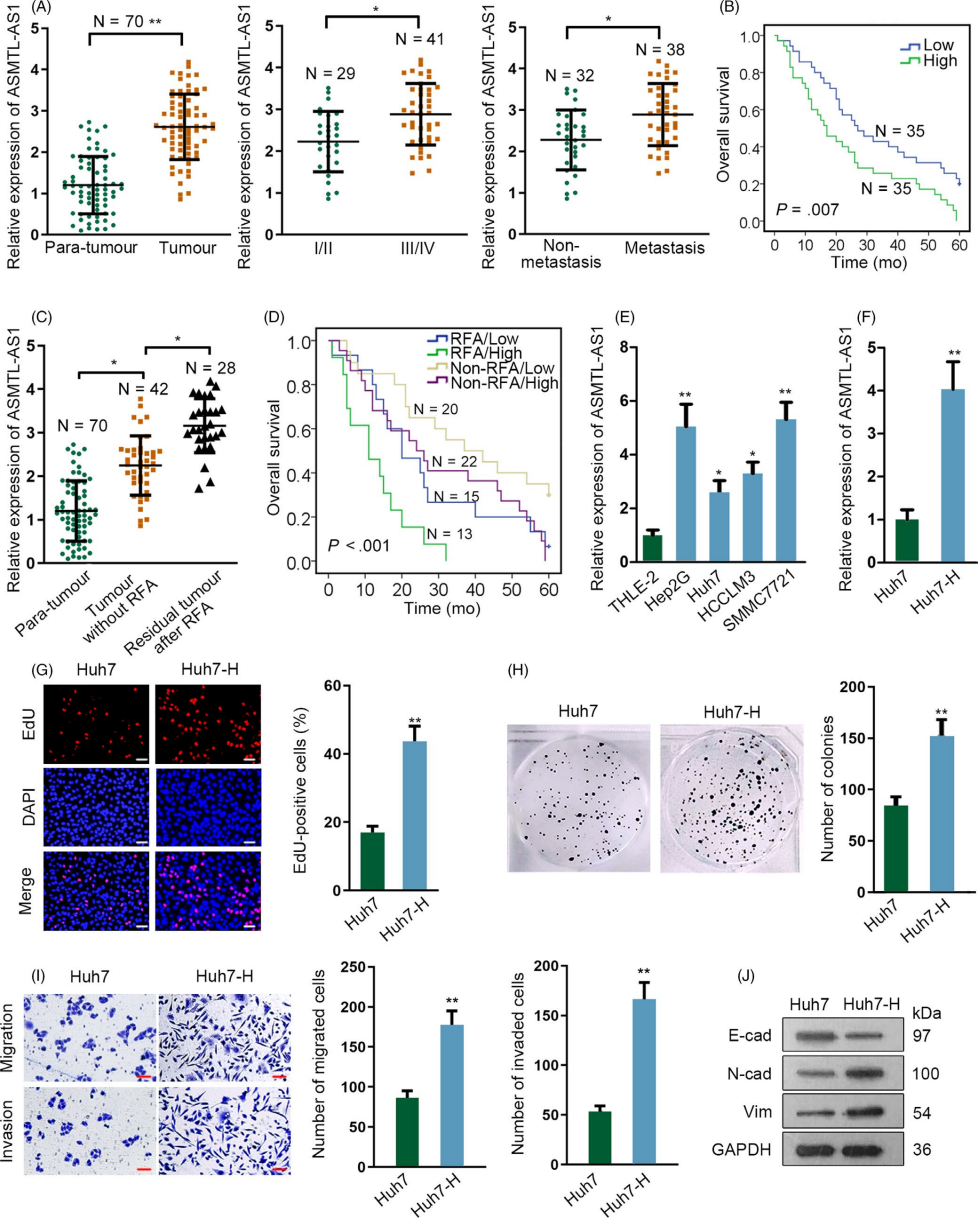
ASMTL‐AS1 was highly expressed in residual HCC after insufficient RFA and Huh7 cells after heating. A, The expression pattern of ASMTL‐AS1 in indicated 70 of HCC tissues was evaluated by qRT‐PCR. B, Kaplan‐Meier curve determined the overall survival of HCC patients with high or low ASMTL‐AS1 level. C, qRT‐PCR result of ASMTL‐AS1 expression in 42 HCC tissues and 28 residual tumours after insufficient RFA, compared to 70 of all para‐tissues. D, Kaplan‐Meier curve assessed the overall survival of HCC patients suffered different treatment according to ASMTL‐AS1 expression. E, ASMTL‐AS1 level in HCC cell lines was analysed via qRT‐PCR. F, qRT‐PCR estimated the expression of ASMTL‐AS1 in Huh7 cells with or without heat treatment. G‐I, Biological behaviour of Huh7 and Huh7‐H cells was detected through conducting EdU assay (G; scale bar = 200 μm), colony formation assay (H) and transwell assay (I; scale bar = 200 μm). Western blot analysis of EMT‐related protein levels in Huh7 and Huh7‐H cells (J). *P < .05, **P < .01

Therefore, we then analysed the correlation of ASMTL‐AS1 with HCC development. As indicated in Figure 1A, we found that the expression level of ASMTL‐AS1 was markedly elevated in HCC tissues compared to adjacent non‐cancerous tissues and its level seemed to be gradually increased along with cancer development and distant metastasis. In the meantime, we also found that the expression of ASMTL‐AS1 in HCC tissues was strongly correlated with tumour size, distant metastasis and TNM stage (Table 1). Besides, patients with higher ASMTL‐AS1 level always underwent worse outcomes (Figure 1B). More interestingly, ASMTL‐AS1 expression was further enhanced in residual HCC tissues collected from patients with insufficient RFA (Figure 1C). Meanwhile, the prognosis of HCC patients who suffered insufficient RFA was further deteriorated when possessing high ASMTL‐AS1 expression (Figure 1D). All these results suggested ASMTL‐AS1 might play an accelerating role in the development of residual HCC after insufficient RFA.

**Table 1:** Relationship between ASMTL‐AS1 expression and several clinical characteristics of HCC patients (n = 70)

Variable	ASMTL‐AS1 expression		*P*‐value
	Low	High	
Gender			
Male	20	18	.810624
Female	15	17	
Age			
≤55	13	15	.807525
>55	22	20	
Cirrhosis			
No	12	10	.797226
Yes	23	25	
Tumour size			
≤5	20	5	.000376
>5	15	30	
Tumour number			
≤3	10	8	.785076
>3	25	27	
TNM stage			
I/II	21	8	.006776
III/IV	14	27	
Distant metastasis			
No	23	9	.001611
Yes	12	26	

Note

Low/high by the sample mean. Pearson chi‐squared test. *P* < .05 was considered to be statistically significant.

To make sure the exact function of ASMTL‐AS1 in residual HCC after insufficient RFA, in vitro experiments were conducted subsequently. It was showed that ASMTL‐AS1 was highly expressed in HCC cell lines compared to normal human liver immortalized cell line THLE‐2 (Figure 1E). Afterwards, Huh7 and HCCLM3 cells processing heat treatment were employed to simulate the residual tumour cells after insufficient RFA in vitro and such cells were then called Huh7‐H and HCCLM3‐H cells, respectively. As revealed by qRT‐PCR, the expression of ASMTL‐AS1 was robustly stimulated after heating (Figure 1F and Figure S2A). Furthermore, such change caused by heating made Huh7‐H cells grow faster and have more invasiveness and migration capability (Figure 1G‐J), so was in HCCLM3‐H cells (Figure S2B). Taken together, ASMTL‐AS1 is significantly up‐regulated in residual HCC tissues after insufficient RFA as well as in heated Huh7 cells.

### 3.2 ASMTL‐AS1 facilitates HCC cell growth and metastasis both in vitro and in vivo

**Figure 2:**
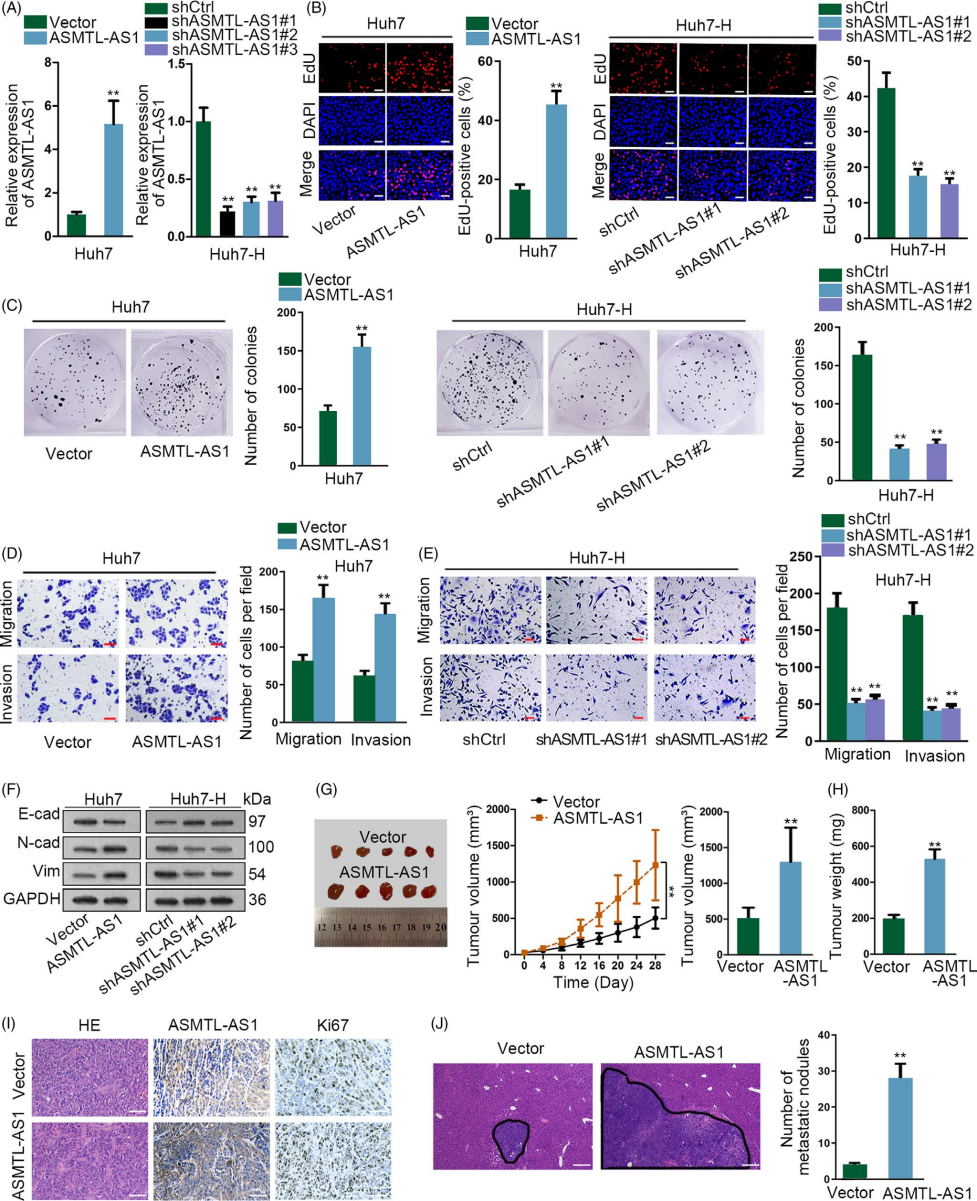
ASMTL‐AS1 facilitated HCC cell growth and metastasis. A, Overexpression or knockdown efficiencies, respectively, in Huh7 or Huh7‐H cells were tested by qRT‐PCR analysis. B‐F, ASMTL‐AS1 function on HCC cell proliferation, motility and EMT was assessed by EdU (B; scale bar = 200 μm), colony formation (C) and transwell assays (D, E; scale bar = 200 μm) as well as Western blot (F), as needed. G, H, In vivo tumour growth experiments showed silencing ASMTL‐AS1 hindered tumour growth in vivo. I, The expression of ASMTL‐AS1 and Ki67 was, respectively, examined by ISH and IHC staining. Scale bar = 200 μm. J, HE staining and corresponding quantification of in vivo metastatic tumours in liver of mice in indicated groups. Scale bar = 400 μm. **P < .01

For the next step, we probed the relationship between ASMTL‐AS1 expression and the malignancy of residual HCC cells. In this situation, gain‐ and loss‐of‐function assays were carried out. It was confirmed that the level of ASMTL‐AS1 was successfully overexpressed in Huh7 cells after transfecting with plasmids containing full length of ASMTL‐AS1 and that it was indeed silenced in Huh7‐H cells under the transfection of three shRNAs targeting ASMTL‐AS1 (Figure 2A). Additionally, Huh7‐H cells with shASMTL‐AS1#1/2 were further applied in subsequent assays considering their better knockdown efficiencies. Resultantly, the proliferative ability of HCC cells was promoted by enhanced ASMTL‐AS1 expression but obviously hindered under ASMTL‐AS1 down‐regulation (Figure 2B,C). Likewise, overexpressed ASMTL‐AS1 apparently strengthened the migratory and invasive capacities in Huh7 cells (Figure 2D), whereas silencing ASMTL‐AS1 led to restrained motility in Huh7‐H cells (Figure 2E). Also, the level of epithelial marker E‐cadherin declined while that of mesenchymal markers N‐cadherin and Vimentin were increased in Huh7 cells responding to ASMTL‐AS1 up‐regulation; however, opposite results were observed in ASMTL‐AS1‐inhibited Huh7‐H cells (Figure 2F). Moreover, similar phenomena were also observed in ASMTL‐AS1‐overexpressed HCCLM3 and ASMTL‐AS1‐depleted HCCLM3‐H cells (Figure S2C‐E). These data demonstrated that ASMTL‐AS1 promotes cell proliferation, migration and invasion as well as EMT in residual HCC cells in vitro.

To provide in vivo evidence, we also performed in vivo experiments here. Results indicated that in contrast to tumours derived from control cells, those from ASMTL‐AS1‐overexpressed Huh7 cells grew much faster and finally had bigger sizes and weights (Figure 2G,H). In addition, tumours originated from Huh7 cells with ectopic ASMTL‐AS1 expression were proved to possess higher ASMTL‐AS1 level and stronger Ki67 staining than those from control group (Figure 2I). In the meantime, the impact of ASMTL‐AS1 on the metastasis of HCC cells was also estimated through conducting in vivo metastatic experiments. As a consequence, more metastatic nodules were generated in the liver of mice injected with ASMTL‐AS1‐overexpressed Huh7 cells relative to that of mice from control group (Figure 2J). Altogether, above results certified that ASMTL‐AS1 can aggravate malignant phenotypes in HCC both in vitro and in vivo.

### 3.3 ASMTL‐AS1 is trans‐activated by MYC in residual HCC cells

**Figure 3:**
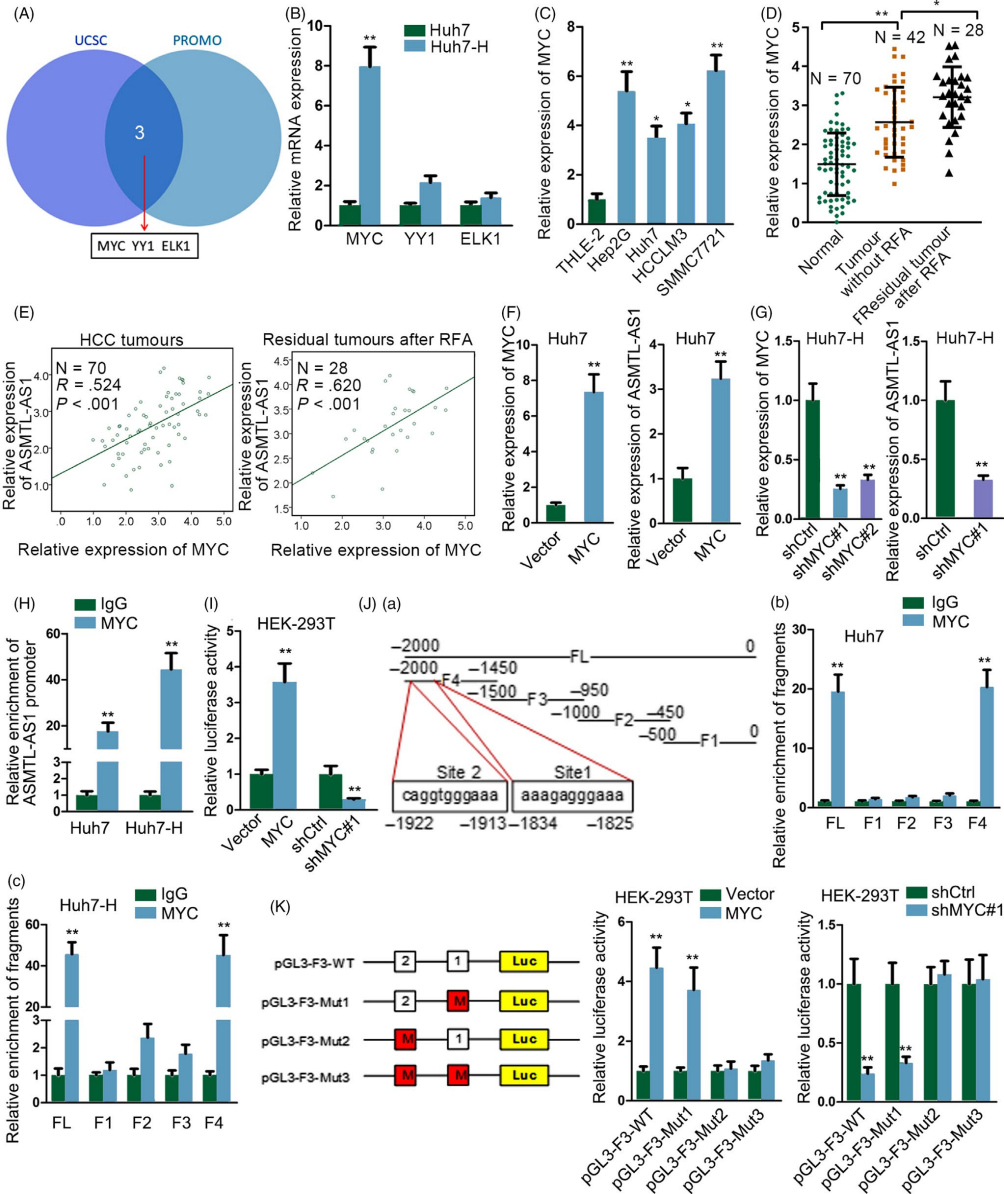
ASMTL‐AS1 was transcriptionally enhanced by MYC. A, Transcription factors for ASMTL‐AS1 were predicted by UCSC and PROMO. B, Relative expression of three potential transcription factors in Huh7 and Huh7‐H cells was tested through qRT‐PCR. C, D, qRT‐PCR results of MYC in HCC cell lines (C) or in indicated HCC tissues (D). E, Pearson's correlation analysis determined the association between MYC and ASMTL‐AS1 in clinical tissues from HCC patients with or without RFA treatment. F, G, qRT‐PCR analysis of the expression levels of MYC or ASMTL‐AS1 in MYC‐overexpressed Huh7 cells or in MYC‐inhibited Huh7‐H cells. H, I, The interaction of MYC with ASMTL‐AS1 promoter and its impact on ASMTL‐AS1 transcription were evaluated via ChIP and luciferase reporter assays, respectively. J, K, ChIP and luciferase reporter assays further confirmed the précised binding sequences for MYC to activate ASMTL‐AS1 transcription. *P < .05, **P < .01

Afterwards, we wanted to know why and how ASMTL‐AS1 was up‐regulated in residual HCC cells after insufficient RFA. According to the prediction of UCSC and PROMO, there were three transcription factors, including MYC, YY1 and ELK1, that might regulate ASMTL‐AS1 expression (Figure 3A). Nevertheless, only the expression of MYC was evidently potentiated after heat treatment (Figure 3B). Besides, MYC expression was generally enhanced in HCC cell lines contrast to THLE‐2 cells (Figure 3C). Also, the level of MYC was markedly augmented in HCC tissues compared to para‐tissues and it was further increased in HCC tissues after insufficient RFA (Figure 3D). Importantly, we discovered a significant positive correlation between MYC and ASMTL‐AS1 expressions in HCC tissues and a higher significance of their correlation in HCC tissues after insufficient RFA (Figure 3E). Also, it was suggested that ASMTL‐AS1 expression was increased or decreased in the wake of MYC up‐regulation or down‐regulation, respectively (Figure 3F,G). Moreover, results of ChIP assay revealed that ASMTL‐AS1 promoter was prominently captured by anti‐MYC but not anti‐IgG (Figure 3H and Figure S2F), while luciferase reporter assay testified that the luciferase activity of ASMTL‐AS1 promoter was revived by MYC overexpression but suppressed under MYC knockdown (Figure 3I). Hence, we found ASMTL‐AS1 transcription was positively modulated by MYC in residual HCC cells.

Thereafter, we explored the precise sequence through which MYC bound to ASMTL‐AS1 promoter. As indicated in Figure 3Ja, full length of ASMTL‐AS1 promoter (FL) was fragmented into four pieces (F1‐F4, with each fragment about 500bp in length). Intriguingly, in both Huh7 and Huh7‐H cells, only F4 had the similar enrichment in anti‐MYC‐immunoprecipitated compounds as FL (Figure 3Jb,c). Based on the prediction by JASPAR (http://jaspar.genereg.net/), there were two probable sites in F4 for MYC binding (Site 1: from −1825 to −1834; Site 2: from −1913 to −1922). And the subsequent luciferase reporter assay confirmed that MYC interacted with ASMTL‐AS1 promoter at Site 2, since the effect of MYC up‐regulation or knockdown on the luciferase activity of ASMTL‐AS1 promoter was deficient when Site 2 was mutated (Figure 3K). In sum, MYC activates ASMTL‐AS1 transcription by binding to ASMTL‐AS1 promoter at the site of −1913~−1922 upstream transcription start site (TSS).

### 3.4 ASMTL‐AS1 prompts NLK expression in HCC cells via miR‐342‐3p

**Figure 4:**
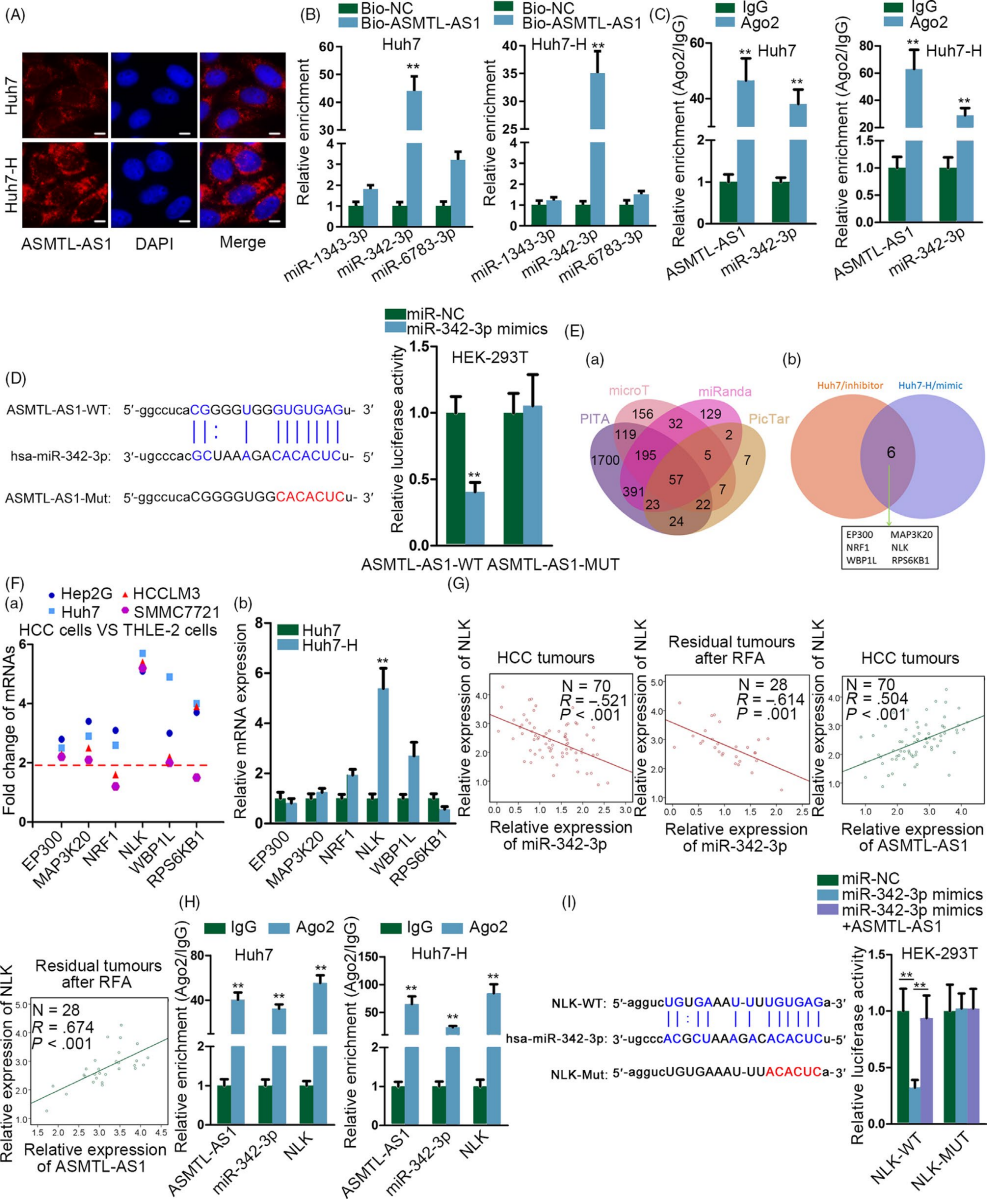
ASMTL‐AS1 elevated the expression of NLK through competitively interacting with miR‐342‐3p. A, Subcellular localization of ASMTL‐AS1 in Huh7 and Huh7‐H cells was validated via FISH. Scale bar = 10 μm. B, RNA pull‐down assay confirmed miR‐342‐3p was sponged by ASMTL‐AS1 in HCC cells. C, RIP assay proved both ASMTL‐AS1 and miR‐342‐3p were enriched in RISC. D, Luciferase reporter assay validated miR‐342‐3p was the downstream of ASMTL‐AS1. E, Targets of miR‐342‐3p were predicted through bioinformatics analysis (a) and further screened out via loss‐ and gain‐of‐miR‐342‐3p analysis (b). F, qRT‐PCR showed the expression of indicated genes in HCC cell lines (a) and in Huh7 cells with or without heating (b). G, The relationship between NLK and either miR‐342‐3p or ASMTL‐AS1 was determined by Pearson's correlation analysis. H, I, RIP and luciferase reporter assays verified the competition between NLK and ASMTL‐AS1 in interacting with miR‐342‐3p‐guided RISCs. **P < .01

In depth, we attempted to identify the detailed mechanism to explain the contribution of ASMTL‐AS1 to the malignancy of residual HCC. The FISH analysis suggested that regardless of heating or not, ASMTL‐AS1 was predominantly present in the cytoplasm of Huh7 and HCCLM3 cells although heat treatment markedly elevated ASMTL‐AS1 signals (Figure 4A and Figure S3A). This result highlighted the potential for ASMTL‐AS1 to function as a competing endogenous RNA (ceRNA) by sponging certain miRNAs. Interestingly, starBase predicted three miRNAs (including miR‐1343‐3p, miR‐342‐3p and miR‐6783‐3p) that might interact with ASMTL‐AS1, among which only miR‐342‐3p was overtly pulled down by Bio‐ASMTL‐AS1 (Figure 4B). Besides, clinical data represented a noticeable reduction of miR‐342‐3p in HCC tissues and its level further declined in residual HCC tissues after insufficient RFA (Figure S3B). Meanwhile, miR‐342‐3p was revealed to be low expressed in HCC cell lines and further down‐regulated after heat treatment (Figure S3C,D). Moreover, we verified that both miR‐342‐3p and ASMTL‐AS1 were harvested by anti‐Ago2 (Figure 4C) and that the luciferase activity of ASMTL‐AS1‐WT was silenced by ectopic expression of miR‐342‐3p (Figure 4D). In this regard, miR‐342‐3p was screened out as the downstream of ASMTL‐AS1 in HCC.

Further, we aimed to find out the downstream effector which was responsible for the regulation of ASMTL‐AS1/miR‐342‐3p axis in the malignant development of residual HCC. Bioinformatics analysis indicated 57 common targets of miR‐342‐3p that predicted by four online tools, such as PITA, microT, miRanda and PicTar (Figure 4Ea). However, only 6 of the 57 targets were shown to be definitely regulated by miR‐342‐3p in both Huh7 and Huh7‐H cells (Figure 4Eb). Additionally, among the 6 targets obtained above, NLK was the only one that was highly expressed in all the four HCC cell lines (Figure 4Fa). Meanwhile, NLK was up‐regulated with the biggest fold change in Huh7‐H cells in comparison with Huh7 cells (Figure 4Fb). Moreover, NLK was negatively correlated with miR‐342‐3p but positively related to ASMTL‐AS1 in all HCC tissues and also in residual HCC tissues after insufficient RFA (Figure 4G). Further, the co‐enrichment of ASMTL‐AS1, miR‐342‐3p and NLK in anti‐Ago2‐induced immunoprecipitate was observed in both Huh7 and Huh7‐H cells (Figure 4H), as well as in HCCLM3 and HCCLM3‐H cells (Figure S3E). Importantly, enhanced expression of miR‐342‐3p impaired the luciferase activity of NLK‐WT, whereas such impairment was reversed by ASMTL‐AS1 overexpression (Figure 4I). Collectively, it could be concluded that ASMTL‐AS1 enhances NLK expression in HCC by secluding miR‐342‐3p.

### 3.5 ASMTL‐AS1 contributes to the translocation of YAP to nucleus of HCC cells through NLK‐dependent way

**Figure 5:**
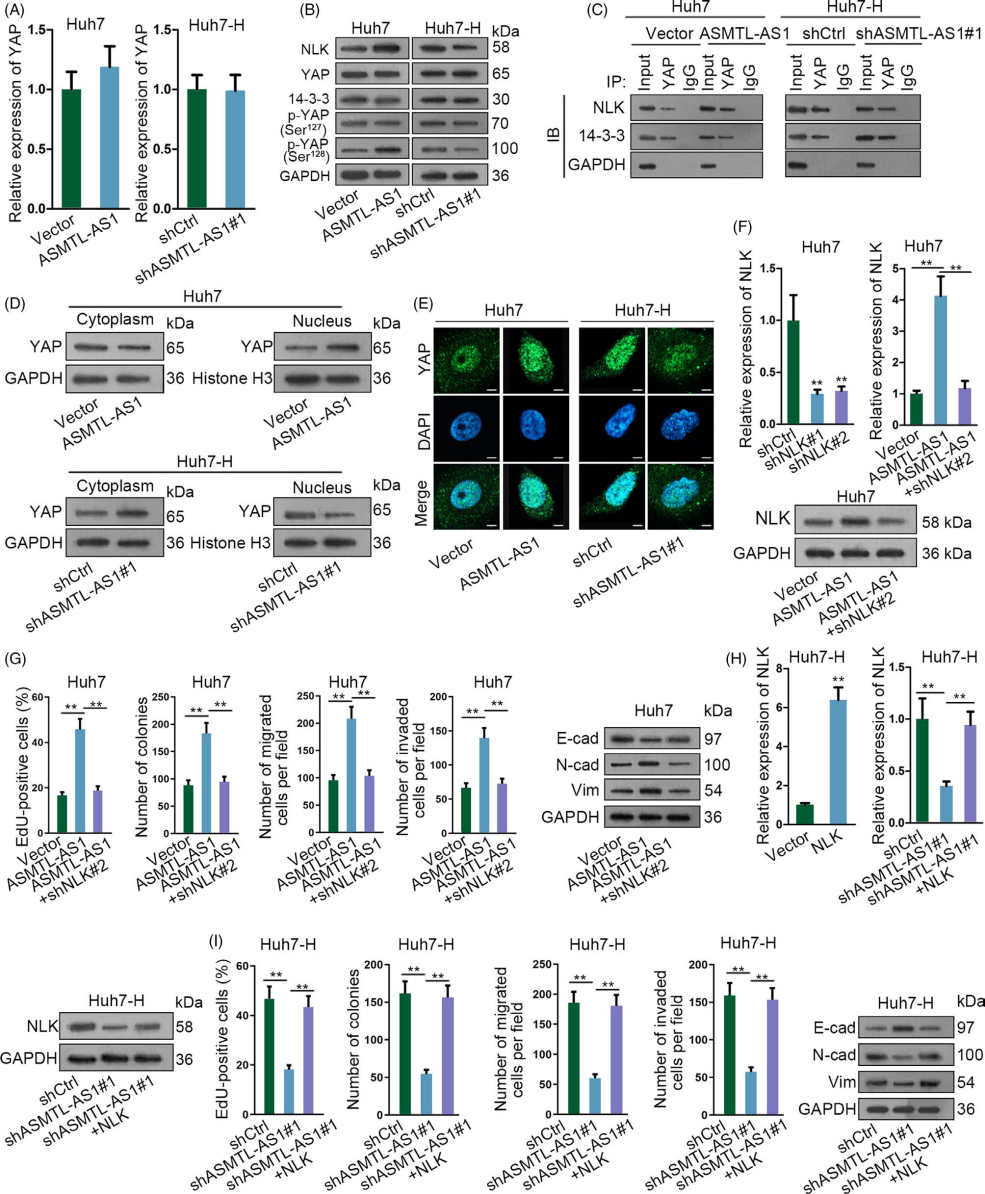
ASMTL‐AS1 regulated HCC malignancy via NLK‐activated YAP signalling. A, qRT‐PCR revealed ASMTL‐AS1 had no impact on the mRNA expression of YAP. B, The protein expression of NLK, YAP and p‐YAP at Ser^127^ or Ser^128^ in ASMTL‐AS1‐overexpressed Huh7 cells or ASMTL‐AS1‐depleted Huh7‐H cells was tested by Western blotting. C, Influence of ASMTL‐AS1 on the interaction of YAP with NLK or 14‐3‐3 was assayed via Co‐IP. D, E, Impact of ASMTL‐AS1 on YAP translocation was assessed by subcellular fractionation plus Western blot (D) and IF assay (E; scale bar = 20 μm). F, The mRNA or protein expression of NLK in indicated cells was analysed by qRT‐PCR or Western blotting, respectively. G, Rescue assays indicated that NLK knockdown recovered ASMTL‐AS1 overexpression‐accelerated proliferation and motility in Huh7 cells. H, I, Rescue assays proved overexpression of NLK countervailed the inhibitory effect of ASMTL‐AS1 depletion on the malignant behaviours of Huh7‐H cells. **P < .01

Previously, NLK has been reported to be involved in the regulation of several signalling pathways.^21^ Since NLK is up‐regulated and plays a tumorigenic part in HCC,^22^, ^23^ here we wondered whether ASMTL‐AS1 functioned in HCC by targeting NLK‐regulated tumour‐suppressive Hippo signalling. Previous study identified that NLK can interact with YAP and then phosphorylate YAP at Ser^128^, leading to reduced YAP interaction with 14‐3‐3 and enhanced nuclear translocation of YAP.^24^ Herein, we planned to make sure whether this mechanism also worked in HCC. As a result, ASMTL‐AS1 had no impact on YAP expression at both mRNA and protein levels; however, the effect of ASMTL‐AS1 on p‐YAP (Ser^128^) was along with its influence on NLK protein expression, whereas p‐YAP (Ser^127^) unchanged all the way (Figure 5A,B and Figure S3F,G). Of note, ASMTL‐AS1 overexpression resulted in enhanced YAP interaction with NLK but blocked YAP binding to 14‐3‐3 in Huh7 and HCCLM3 cells, whereas ASMTL‐AS1 inhibition in Huh7‐H and HCCLM3‐H cells gave rise to the opposite way (Figure 5C and Figure S3H). Further, such changes made it easier for YAP to translocate to nucleus under ASMTL‐AS1 up‐regulation but harder upon ASMTL‐AS1 depletion (Figure 5D,E). Collectively, ASMTL‐AS1 facilitates the nuclear translocation of YAP in HCC cells by NLK‐mediated manner.

### 3.6 ASMTL‐AS1 aggravates the malignancy of HCC cells through targeting NLK

In subsequence, we probed whether the facilitating role of ASMTL‐AS1 in the malignancy of HCC was mediated by NLK. As proved in Figure 5F, both the mRNA and protein expressions of NLK that were increased in ASMTL‐AS1‐up‐regulated Huh7 cells were normalized in response to NLK knockdown. Moreover, such normalization on NLK expression led to recovered cell proliferation, motility and EMT in Huh7 cells with ASMTL‐AS1 overexpression (Figure 5G). On the contrary, forced NLK expression counteracted the impact of ASMTL‐AS1 suppression on the biological behaviours of Huh7‐H cells (Figure 5H,I). All in all, these data illustrated that ASMTL‐AS1 accelerates the malignant phenotypes of HCC cells by NLK‐relied pathway.

### 3.7 Exosome‐transmitted ASMTL‐AS1 confers malignant behaviours between residual HCC cells through NLK/YAP signalling

**Figure 6:**
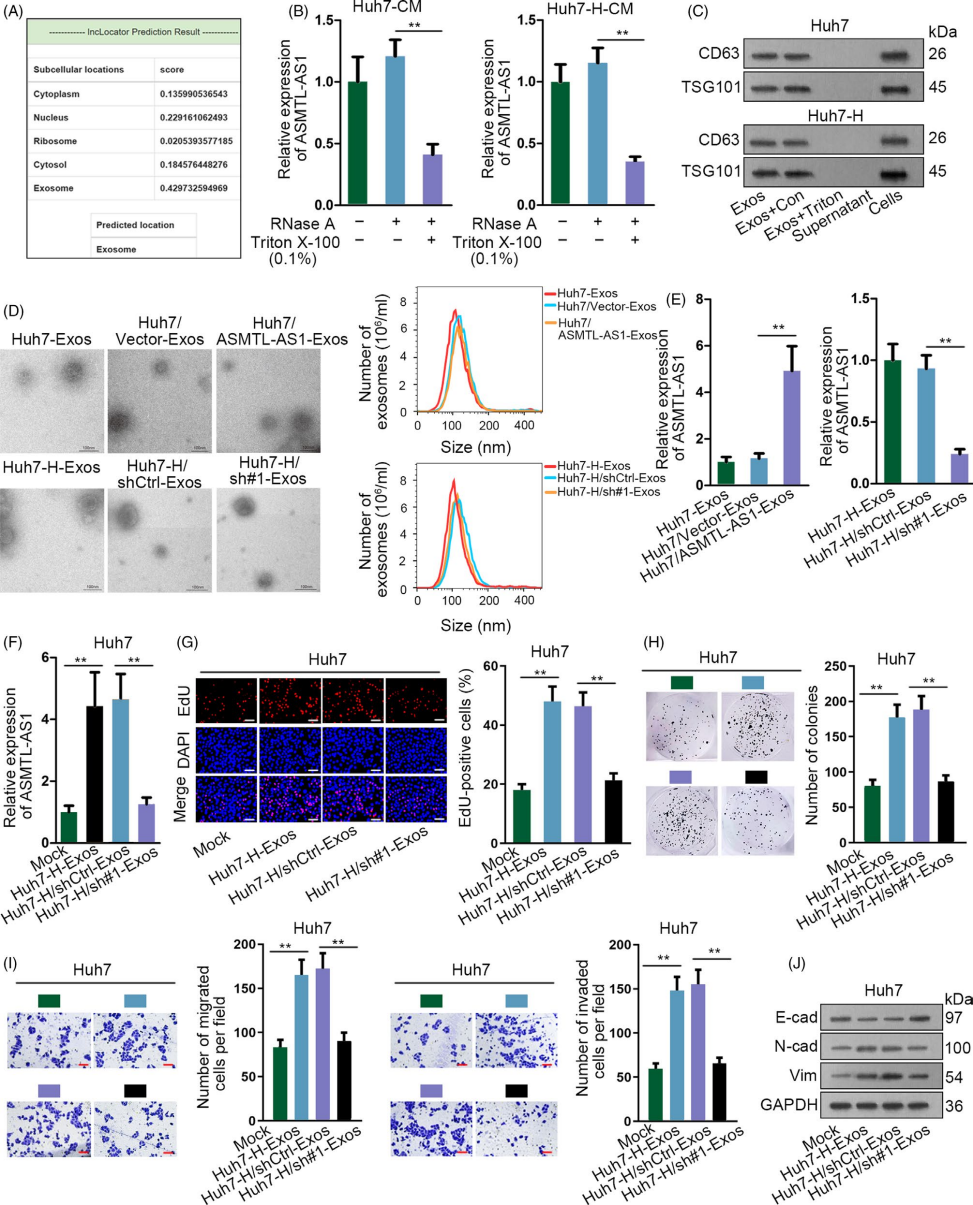
Exosome‐transferred ASMTL‐AS1 delivered malignancies between HCC cells. A, Online tool lncLocator predicted that ASMTL‐AS1 mainly located in exosomes. B, ASMTL‐AS1 level in Huh7 and Huh7‐H under diverse treatments was estimated by qRT‐PCR. C, Western blot identified exosomes through two markers including CD63 and TSG101. D, Representative images of exosomes from indicated cells were captured by electron microscopy, and the size and number of these exosomes were determined via NanoSight particle tracking analysis. Scale bar = 100 nm. E, The level of ASMTL‐AS1 in above exosomes was analysed through qRT‐PCR. F‐J, Impact of exosomes from control or ASMTL‐AS1‐silenced Huh7‐H cells on the cellular processes of Huh7 cells was estimated by EdU assay (scale bar = 200 μm), colony formation assay, transwell assay (scale bar = 200 μm) and Western blot. **P < .01

Recently, the importance of tumour microenvironment has been highlighted in cancer development. Exosomes are a subset of extracellular vesicles that exist in tumour microenvironment to confer malignant information to recipient cells.^25^ Interestingly, the online lncLocator indicated that ASMTL‐AS1 was largely in the exosomes of cells (Figure 6A). Besides, it was revealed that the expression of ASMTL‐AS1 in the culture medium (CM) of Huh7 and Huh7‐H cells was unaffected by sole treatment with RNase A but was largely abolished after the co‐treatment of RNase A and Triton X‐100 (Figure 6B), suggesting that extracellular ASMTL‐AS1 did not exist as a released form but was protected through being wrapped by membrane. Subsequently, exosomes was extracted and purified from CM and further identified via exosome markers CD63 and TSG101 (Figure 6C). More importantly, we proved that there were no significant differences in the size and number of exosomes between Huh7 and Huh7‐H cells (Figure 6D). Furthermore, ASMTL‐AS1 expression in exosomes was altered in line with its level in whole cells (Figure 6E).

Next, we launched an investigation into the function of exosomes containing ASMTL‐AS1 in HCC. As shown in Figure 6F, the intracellular level of ASMTL‐AS1 in Huh7 cells was remarkably stimulated under the incubation with exosomes collected from CM of Huh7‐H cells (Huh7‐H‐Exos), whereas it was reverted to normal when being incubated with exosomes from CM of ASMTL‐AS1‐silenced Huh7‐H cells (Huh7‐H/sh#1‐Exos). More intriguingly, incubation of Huh7‐H‐Exos accelerated cell proliferation, migration and invasion as well as EMT in Huh7 cells, while such promotion by exosomes from Huh7‐H cells could be abated by ASMTL‐AS1 depletion in Huh7‐H cells (Figure 6G‐J). In addition, we observed opposite phenomena in Huh7‐H cells when incubated with exosomes from CM of Huh7 cells under diverse conditions (Figure S4A‐F). Of importance, the expression of NLK and p‐YAP (Ser^128^) as well as YAP nuclear translocation in HCC cells was positively changed with ASMTL‐AS1 expression transmitted by exosomes, whereas total YAP and p‐YAP (Ser^127^) scarcely altered all the time (Figure S4G‐J). To be concluded, ASMTL‐AS1 can be transmitted by exosomes to deliver malignancy to recipient HCC cells by targeting NLK/YAP axis.

### 3.8 Exosomal ASMTL‐AS1 contributes to the malignancy of residual HCC after insufficient RFA through NLK/YAP axis

**Figure 7:**
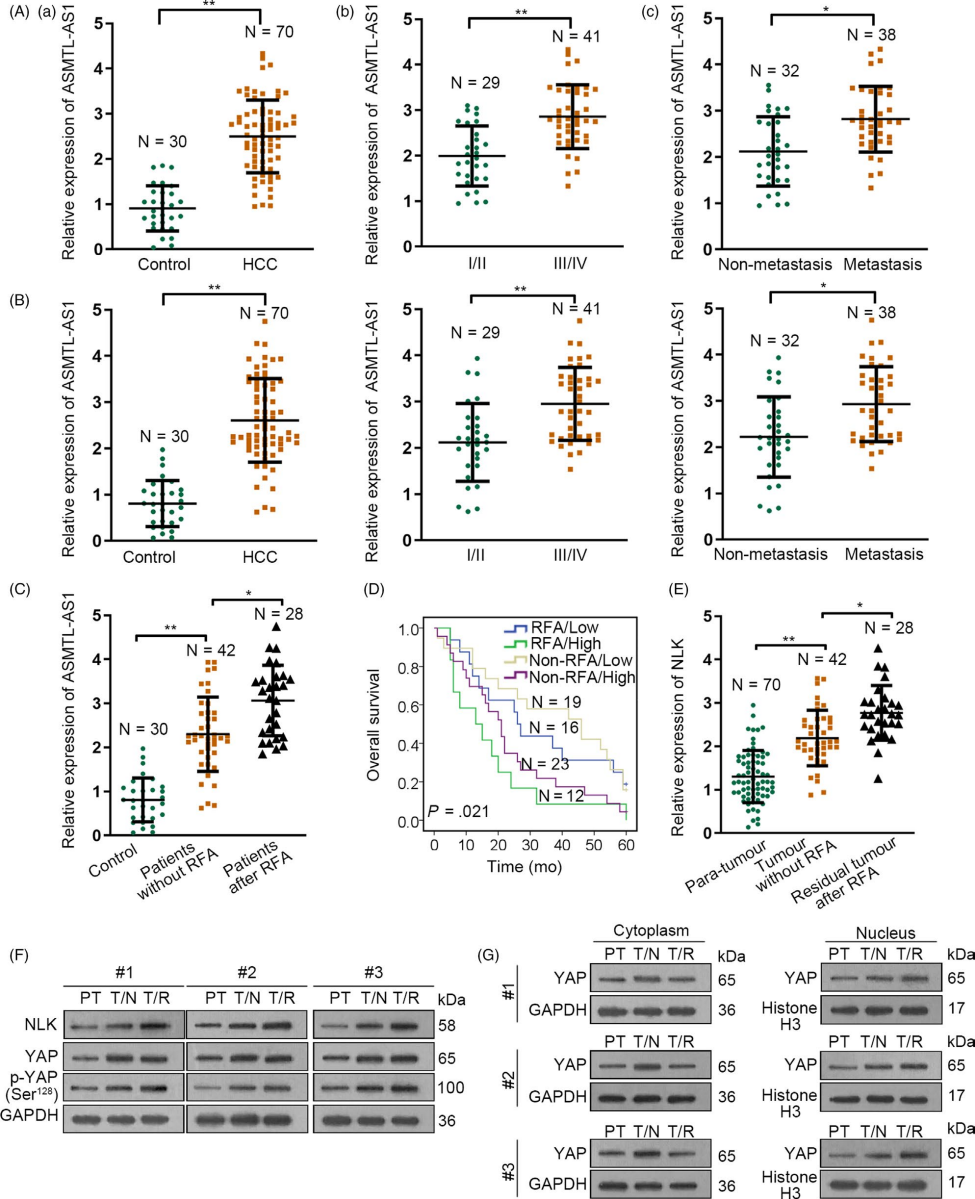
ASMTL‐AS1/NLK/YAP axis in clinical HCC patients. A‐C, qRT‐PCR analysis results of ASMTL‐AS1 expression in different groups of HCC patients: (A) The expression of ASMTL‐AS1 in the serum of indicated HCC patients (control referred to serums from 30 healthy individuals); (B) ASMTL‐AS1 expression in serum exosomes (SEs) of above HCC patients; and (C) ASMTL‐AS1 expression in SEs of HCC patients with or without RFA treatment. D, Overall survival rates of four indicated kinds of HCC patients were assessed through Kaplan‐Meier analysis. E, qRT‐PCR revealed the level of NLK in para‐tumours, primary HCC tumours without RFA treatment and residual HCC tumours after insufficient RFA. F, The levels of indicated proteins in above three kinds of tumours were analysed by Western blot. G, YAP localization in above tumours was also determined through Western blot. PT meant para‐carcinoma tissues. T/N referred to HCC tissues from patients with no RFA treatment, and T/R indicated HCC tissues from patients with insufficient RFA. *P < .05, **P < .01

To further make sure the role of ASMTL‐AS1/miR‐342‐3p/NLK/YAP axis in residual HCC after insufficient RFA, we analysed the expression of relevant genes in the serum and serum exosomes (SEs) from HCC patients including those with insufficient RFA. Results exhibited that compared to the serum of healthy controls, ASMTL‐AS1 was highly expressed in HCC patients (Figure 7Aa). Meanwhile, the gradual increasing tendency of ASMTL‐AS1 was revealed in serums from HCC patients along with the progress of disease stage (Figure 7Ab). Likewise, the expression of ASMTL‐AS1 was much higher in the serum of HCC patients with metastasis than those without (Figure 7Ac). Consistently, similar trends of ASMTL‐AS1 expression were also observed in SEs of HCC patients (Figure 7B). Moreover, it was disclosed that ASMTL‐AS1 expression was further reinforced in SEs of patients who had residual HCC after insufficient RFA (Figure 7C). More importantly, although high exosomal ASMTL‐AS1 expression resulted in disappointing survival rates in HCC patients without RFA treatment, it gave rise to worse outcomes in those with insufficient RFA (Figure 7D). Additionally, we found NLK expression continuously augmented, just as the trend of ASMTL‐AS1 in different tissues obtained from HCC patients (Figure 7E). As a result, more YAP was phosphorylated at Ser^128^ and therefore induced more YAP trans‐locating to nucleus in tumours from patients after insufficient RFA than in those from patients without RFA due to higher NLK protein (Figure 7F,G). On the whole, ASMTL‐AS1 transferred by exosomes enhances the malignancy of residual HCC tumours after insufficient RFA through NLK/YAP signalling.

## 4 DISCUSSION

Presently, RFA has been regarded as the main local treatment for patients advanced HCC who are not suitable for surgical resection.^26^ Nevertheless, insufficient RFA leads to promoted aggressiveness in residual HCC cells, resulting in high rate of recurrence and rapid progression in residual tumour.^27^ Over the decades, lncRNAs have been largely proposed as critical molecules that are implicated in regulating cancer development.^28^ In current study, we explored a novel lncRNA ASMTL‐AS1 in residual HCC after insufficient RFA. We found ASMTL‐AS1 up‐regulation in HCC tissues was strongly associated with advanced staged and metastasis, which implied the correlation of ASMTL‐AS1 expression with malignancy in HCC. Moreover, a higher level of ASMTL‐AS1 in residual tumours after insufficient RFA was proved. Further, here we also clarified the relationship between ASMTL‐AS1 level and prognosis of HCC patients with or without RFA treatment. Subsequently, we established in vitro insufficient RFA model in Huh7 cells by using heat treatment. Firstly, we confirmed that heat treatment made Huh7 cells express higher ASMTL‐AS1 and become more malignant. Also, ASMTL‐AS1 was explained as a contributor to the malignant phenotypes of both Huh7 and Huh7‐H cells, opposite to the role of lncRNA FUNDC2P4 in residual HCC after insufficient RFA.^15^ Furthermore, we identified that ASMTL‐AS1 up‐regulation was attributed to the transcriptional activation by MYC, a well‐recognized transcription factor that is always up‐regulated in multiple cancers including HCC.^29^

Interestingly, the function of lncRNAs usually links with their subcellular fates.^30^ For example, lncRNA SNHG1 was retented in nucleus and competes with p53 to interact with hnRNPC.^31^ LncRNA CRCMSL prevents the nucleocytoplasmic shuttling of HMGB2 by directly binding to HMGB2 in the cytoplasm.^32^ In this study, we discovered that ASMTL‐AS1 mainly existed in the cytoplasm of HCC cells, regardless of heat treatment. Recently, a ceRNA network guided by lncRNAs has increasingly emerged as crucial molecular mechanism underlying carcinogenesis.^33^ Here, we revealed that miR‐342‐3p, an already identified tumour suppressor in HCC,^34^, ^35^ was sponged by ASMTL‐AS1 and its expression was negatively associated with ASMTL‐AS1 level in HCC tissues as well as residual tumours after insufficient RFA.

NLK, a gene that has been verified as an oncogene in HCC,^22^ was further screened out to be the downstream responsible factor for ASMTL‐AS1/miR‐342‐3p axis. Besides, NLK is reported to regulate various signalling pathways, such as pro‐tumour Wnt,^36^ Notch^37^ and mTOR signalling^38^ as well as anti‐tumour Hippo signalling.^39^ This might be the reason why NLK exerts diverse functions during different cancers.^23^, ^40^, ^41^ However, sometimes there are contradictions about NLK function when in different diseases. For instance, Masoumi, KC et al demonstrated that NLK negatively regulates Wnt signalling by phosphorylating HDAC1.^42^ However, Jung et al^22^ also found that NLK silence led to reduced β‐catenin expression, suggesting a positive regulation of NLK on Wnt pathway. Thus, considering the tumourigenic role of NLK in HCC, we focused on its impact on Hippo pathway and confirmed that ASMTL‐AS1 promoted YAP nuclear translocation by regulating miR‐342‐3p/NLK signalling currently.

Meanwhile, here we unveiled a surprising discovery that ASMTL‐AS1 could be packaged into exosomes and subsequently relay between HCC cells. During the past few decades, exosomes have been testified to play key roles in cancer development due to their ability to convey information to recipient cells.^43^ In our research, we certified exosomes could affect the biological behaviours of Huh7 and Huh7‐H cells depending on ASMTL‐AS1 expression they contained to some extent. More importantly, cell received exosomes with high or low ASMTL‐AS1 level could finally affect NLK expression and therefore influence YAP nuclear translocation, which was validated in vitro and further evidenced by clinical data.

In conclusion, this research illustrated the participation of an ASMTL‐AS1/miR‐342‐3p/NLK/YAP signalling in reinforcing the malignancy of HCC cells, especially those in residual tumours after insufficient RFA. Also, we detected ASMTL‐AS1 could be transmitted between HCC cells to activate such signalling in the recipient cells as well. These findings provide ASMTL‐AS1 as a novel target that is potentially effective for the treatment of HCC patients, especially those after insufficient RFA. Moreover, it also suggests ASMTL‐AS1 may be helpful to prevent the recurrence and metastasis of residual HCC after RFA.

## CONFLICT OF INTEREST

All authors ensure no conflicts of interest in our work.

## AUTHOR CONTRIBUTION

Dening Ma and Xinyi Gao contributed the original article and experiment designing. Zhuo Liu and Xingang Lu devoted to the data, analysis and investigation with the assistance of Haixing Ju and Ning Zhang. All authors approved final manuscript.

## Supporting information


**Figure S1**

**Figure S2**

**Figure S3**

**Figure S4**

## Data Availability

Research data are not shared.
