# Supplementary figures and images for: Exosome‐transferred long non‐coding RNA ASMTL‐AS1 contributes to malignant phenotypes in residual hepatocellular carcinoma after insufficient radiofrequency ablation
Source: Cell Prolif. 2020 Jul 28;53(9):e12795. doi: 10.1111/cpr.12795 (PMC7507479; doi:10.1111/cpr.12795)

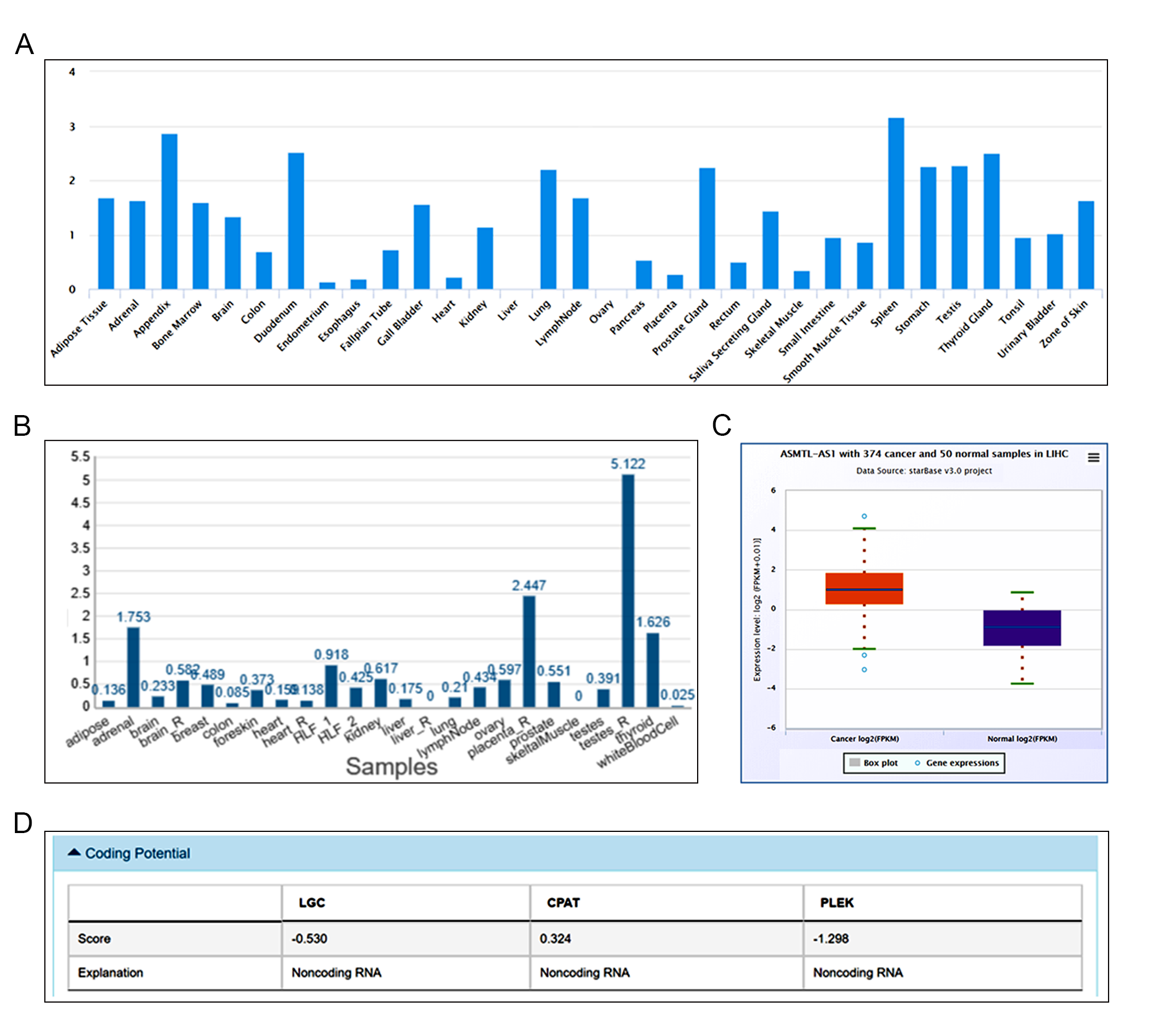

Supplement: Supplementary file 1 — Figure S1 [file CPR-53-e12795-s001.tif]

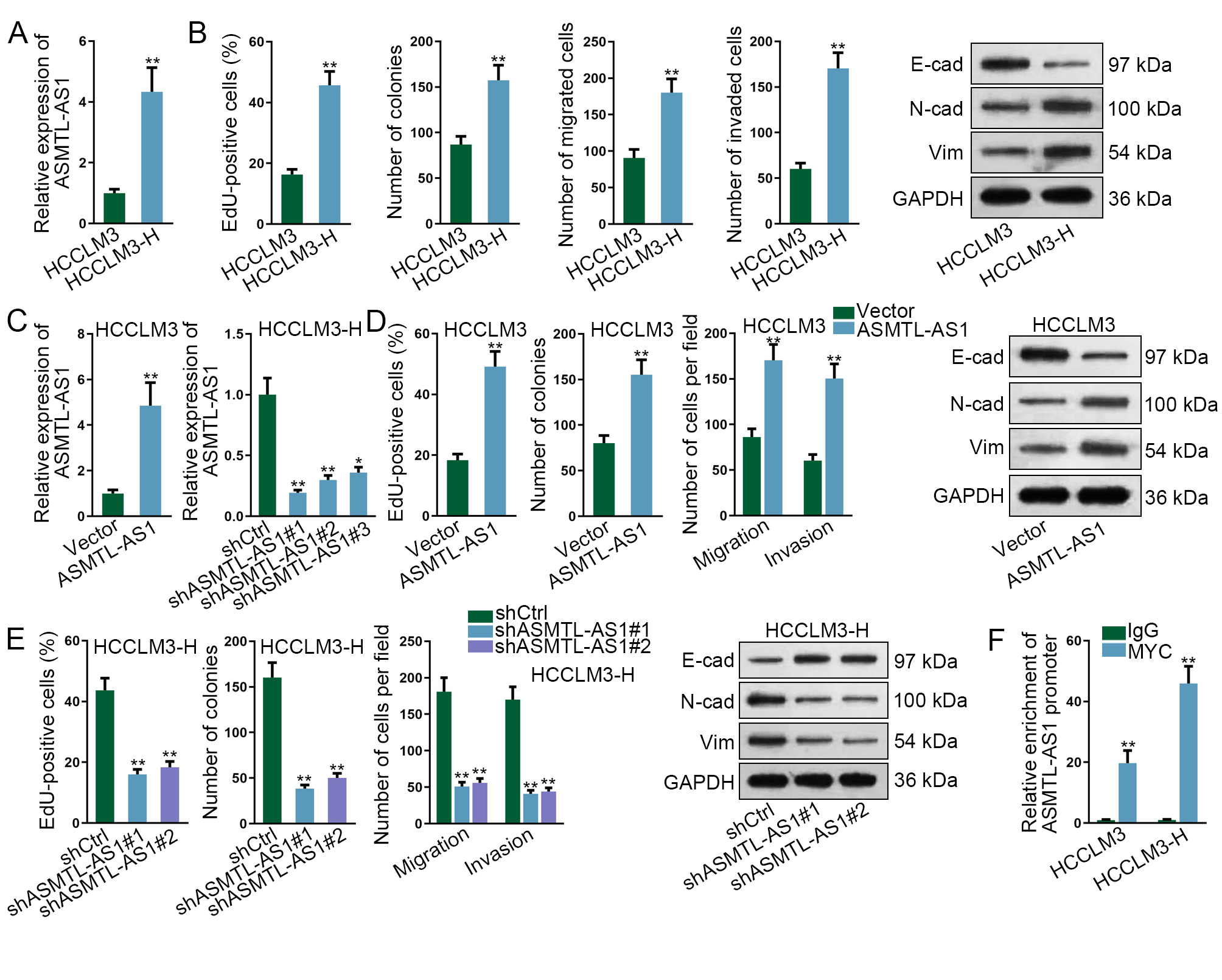

Supplement: Supplementary file 2 — Figure S2 [file CPR-53-e12795-s002.tif]

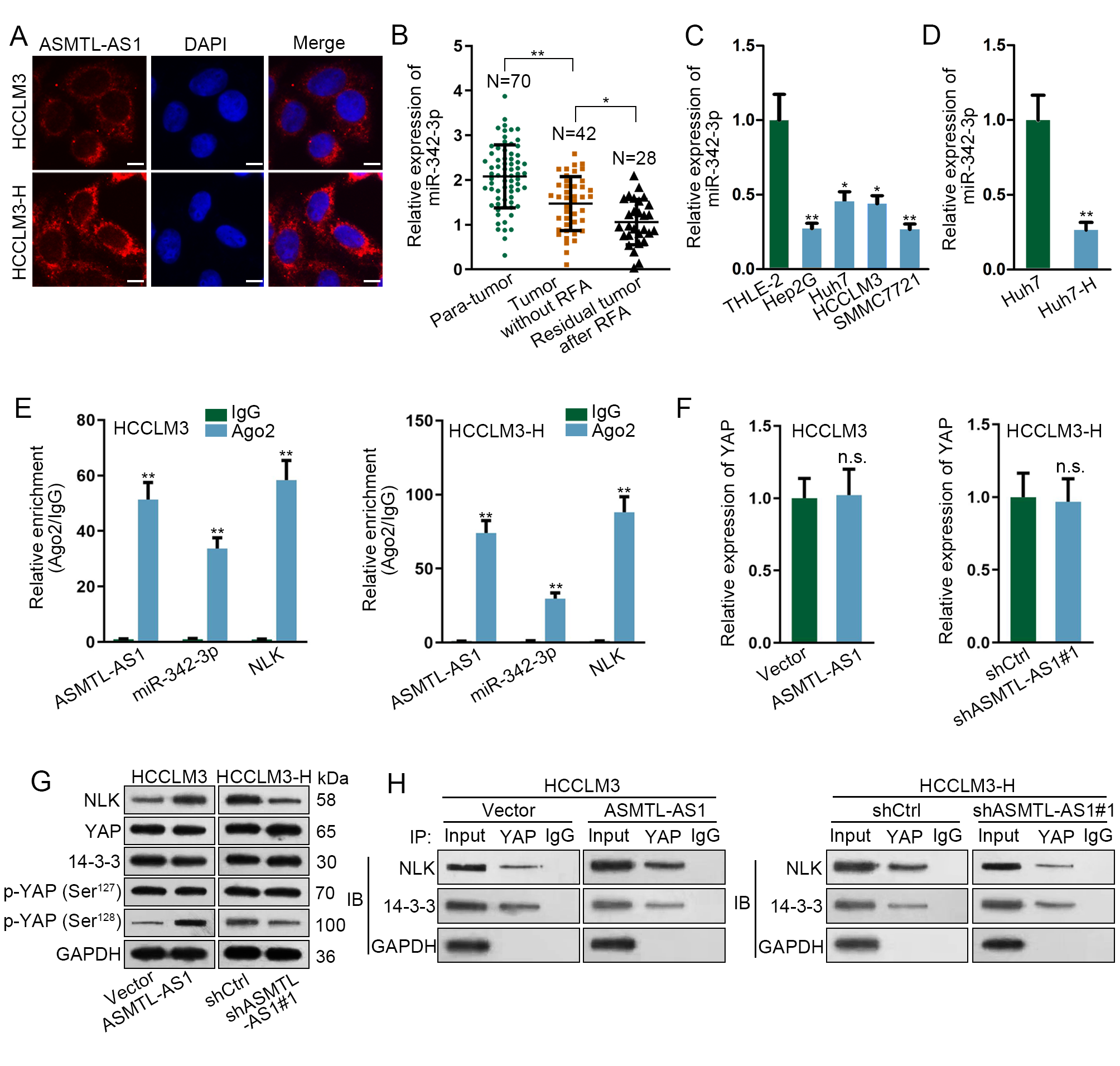

Supplement: Supplementary file 3 — Figure S3 [file CPR-53-e12795-s003.tif]

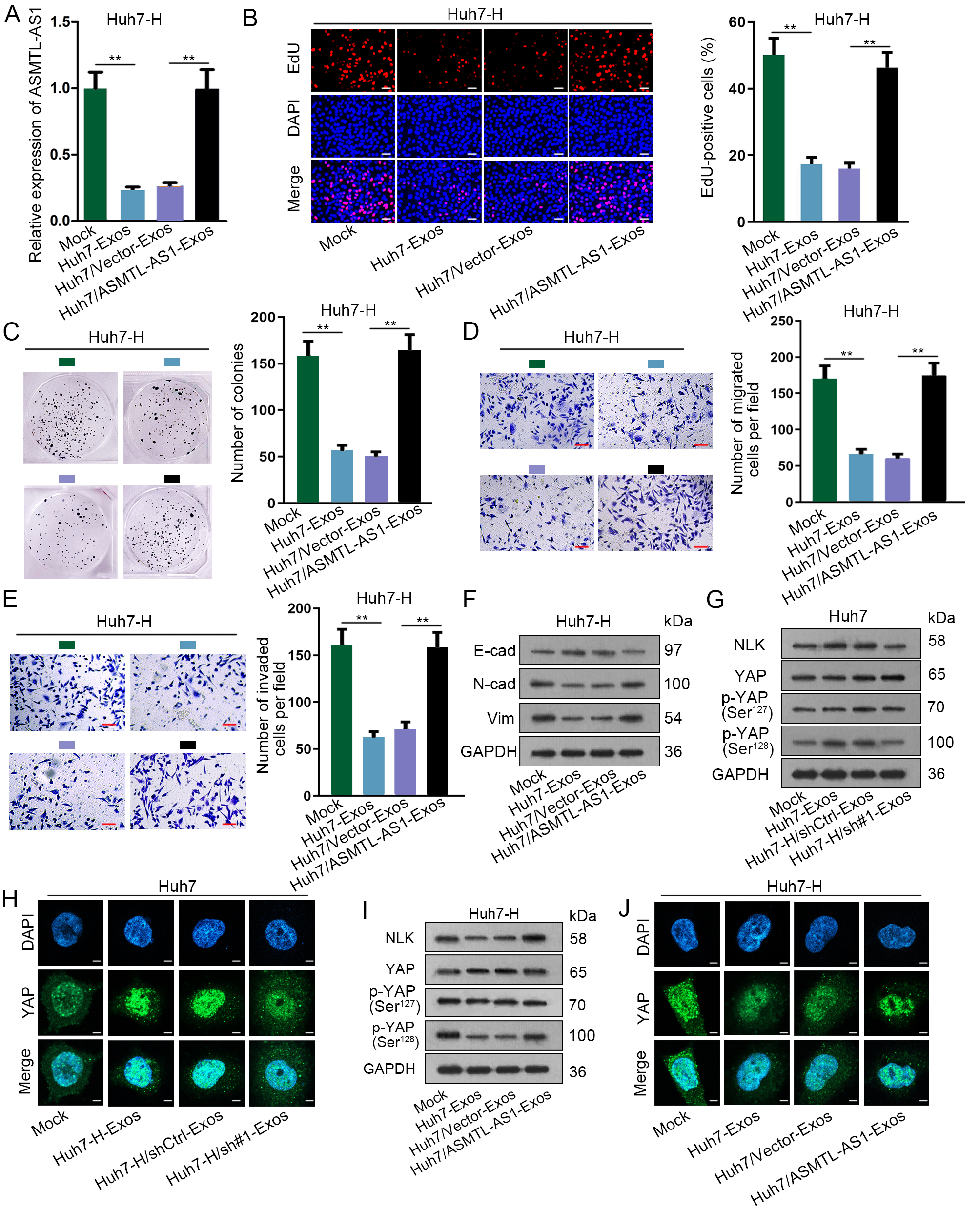

Supplement: Supplementary file 4 — Figure S4 [file CPR-53-e12795-s004.tif]
